# Direct detection of the myosin super-relaxed state and interacting-heads motif in solution

**DOI:** 10.1016/j.jbc.2021.101157

**Published:** 2021-09-02

**Authors:** Sami Chu, Joseph M. Muretta, David D. Thomas

**Affiliations:** Department of Biochemistry, Molecular Biology, and Biophysics, University of Minnesota, Minneapolis, Minnesota, USA

**Keywords:** myosin, fluorescence resonance energy transfer (FRET), structure–function, enzyme structure, cardiac muscle, HMM, heavy meromyosin, IHM, interacting-heads motif, SRX, super-relaxed state, TR-FRET, time-resolved fluorescence resonance energy transfer

## Abstract

The interacting-heads motif (IHM) is a structure of myosin that has been proposed to modulate cardiac output by occluding myosin molecules from undergoing the force-generating cycle. It is hypothesized to be the structural basis for the super-relaxed state (SRX), a low-ATPase kinetic state thought to be cardioprotective. The goal of the present study was to test this hypothesis by determining directly and quantitatively the fractions of myosin in the IHM and SRX under the same conditions in solution. To detect the structural IHM, we used time-resolved fluorescence resonance energy transfer to quantitate two distinct populations. One population was observed at a center distance of 2.0 nm, whereas the other was not detectable by fluorescence resonance energy transfer, implying a distance greater than 4 nm. We confirmed the IHM assignment to the 2.0-nm population by applying the same cross-linking protocol used previously to image the IHM by electron microscopy. Under the same conditions, we also measured the fraction of myosin in the SRX using stopped-flow kinetics. Our results show that the populations of SRX and IHM myosin were similar, unless treated with mavacamten, a drug that recently completed phase III clinical trials to treat hypertrophic cardiomyopathy and is proposed to act by stabilizing both the SRX and IHM. However, we found that mavacamten had a much greater effect on the SRX (55% increase) than on the IHM (4% increase). We conclude that the IHM structure is sufficient but not necessary to produce the SRX kinetic state.

Myosin II is a motor protein that powers the contraction of muscle through large structural changes when its ATPase activity is activated by actin (1). It consists of a dimer of heavy chain molecules, each binding two light chains, the essential light chain, and regulatory light chain (RLC). In striated muscles (skeletal and cardiac), myosin polymerizes to form bipolar thick filaments through coiled-coil interactions of the C-terminal tail region of the heavy chain. The N-terminal region branches from the tail to form two heads, each containing a globular catalytic domain and a light-chain domain that binds essential light chain and RLC (Fig. 1*A*).

**Figure 1:**
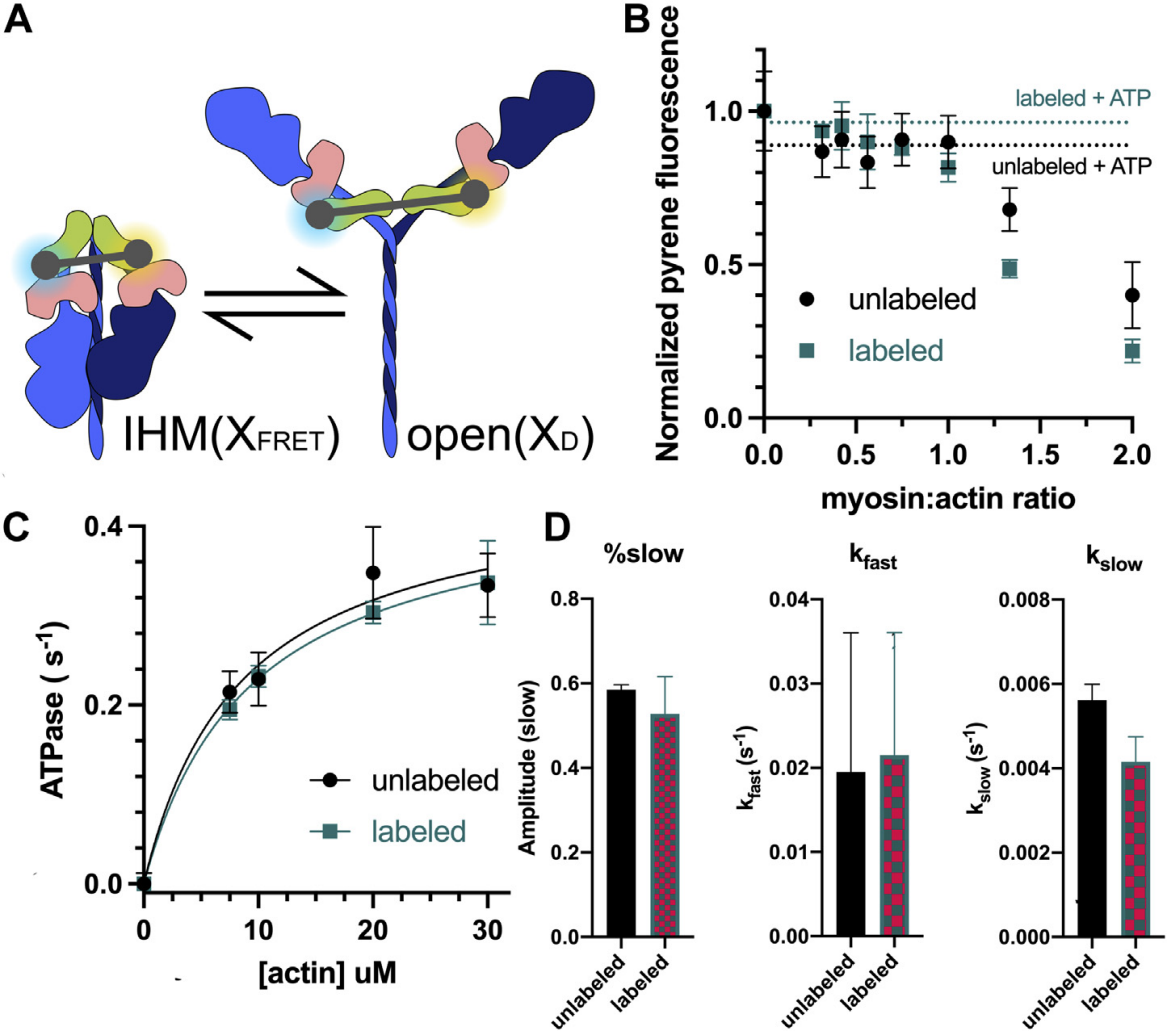
**Labels on HMM have no effect on myosin function.***A*, schematic representation of IHM and 6S myosin molecule with FRET labels attached on the regulatory light chain. Heavy chain is in *blue*, with the blocked head of the IHM in *dark blue* and free head in *light blue*; regulatory light chain is in *green*, and essential light chain is in *pink*. FRET donor (EDANS) is in *blue*, and FRET acceptor (dabcyl) is *orange*. *B*, actin-binding function of labeled HMM measured by fluorescence quenching of pyrene-labeled actin. *Dots* indicated pyrene-labeled actin fluorescence after the addition of labeled or unlabeled HMM in the specified myosin:actin ratio in the absence of ATP. *Dotted line* indicates fluorescence after the addition of 2 mM ATP. (n = 4 per condition, *p* = 0.90 in two-tailed unpaired *t* test). *C*, steady-state ATPase activity of labeled HMM measured by NADH-coupled ATPase activity (n = 3 per condition, *p* = 0.75 in two-tailed unpaired *t* test). *D*, basal ATP turnover measured with and without labels (n = 3 per condition). FRET, fluorescence resonance energy transfer; HMM, heavy meromyosin; IHM, interacting-heads motif.

The regulation of muscle contraction involves primarily the calcium-dependent regulation of the actin-containing thin filament. However, an additional regulatory mechanism has recently come to light, in which myosin regulates itself through autoinhibitory interactions between its two heads. It is proposed that the structural basis for this autoinhibited state is the interacting-heads motif (IHM) (2, 3, 4, 5). Evidence for the IHM has been obtained in several isoforms of myosin from several species through electron microscopy (3, 5, 6, 7), and evidence consistent with it has been observed by X-ray diffraction (8, 9) and fluorescence polarization (10, 11). In the IHM, the catalytic domains of the myosin dimer interact asymmetrically—the converter domain of the free head blocks the actin-binding region of the blocked head (3). Another region of interest in the IHM is the mesa region of the catalytic domain, which is proposed to interact with the portion of the tail region closest to the catalytic domain (also called subfragment-2 or S2) (12, 13). Studies have shown that disease-related mutations in this region decrease the affinity of the catalytic domain for S2, thus destabilizing the IHM (13). The present study uses a cleaved two-headed version of myosin, heavy meromyosin (HMM), which includes the S2 portion of the tail that stabilizes the dimer but lacks the backbone and other accessory proteins that may further stabilize the IHM.

It has been hypothesized that the IHM is the structural basis for the super-relaxed state (SRX), a functional state characterized by extremely slow ATP turnover, 100 times slower than during contraction, and three to ten times slower than is typical for most of the myosin molecules in relaxed muscle (14, 15). SRX is detected biochemically in single-turnover experiments, measuring the decrease in fluorescence that occurs when fluorescent mant-ATP is hydrolyzed, released from myosin, and displaced by nonfluorescent ATP, resulting in a multiple exponential decay, in which the slowest rate (longest lifetime τ) is assigned to the SRX state (14). This kinetic signature has also been observed in isolated myosin molecules and fragments (16). It is generally assumed that the SRX biochemical kinetic state corresponds to the IHM structural state, but this has not been tested directly on the same sample under similar conditions.

To bridge this gap, we have used fluorescence resonance energy transfer (FRET) between the two heads of myosin to detect the IHM structural state directly in solution and directly compare the fraction of myosin molecules in SRX measured by ATP turnover, under similar conditions. In contrast to intensity-based FRET measurement, time-resolved FRET can resolve multiple distinct populations of protein conformations, each giving rise to a distinct lifetime τ of decay after an exciting pulse. To verify that FRET is detecting the IHM, we used a chemical cross-linker that is known to stabilize the IHM.

To further evaluate the relationship between IHM and SRX, we compared how both the fluorescent nucleotide turnover and the structural FRET measurement were affected by mavacamten, a small molecule that has completed phase III clinical trials for treating hypertrophic cardiomyopathy and has been shown to increase the SRX population (16, 17). Given the current state of knowledge, we expected that mavacamten would increase FRET by increasing the fraction of myosin in IHM. We also tested a range of ionic strength shown to affect the SRX population to further probe the relationship between the IHM and SRX (16).

## Results

### FRET labels have no effect on myosin function in the presence of actin

**Figure 2:**
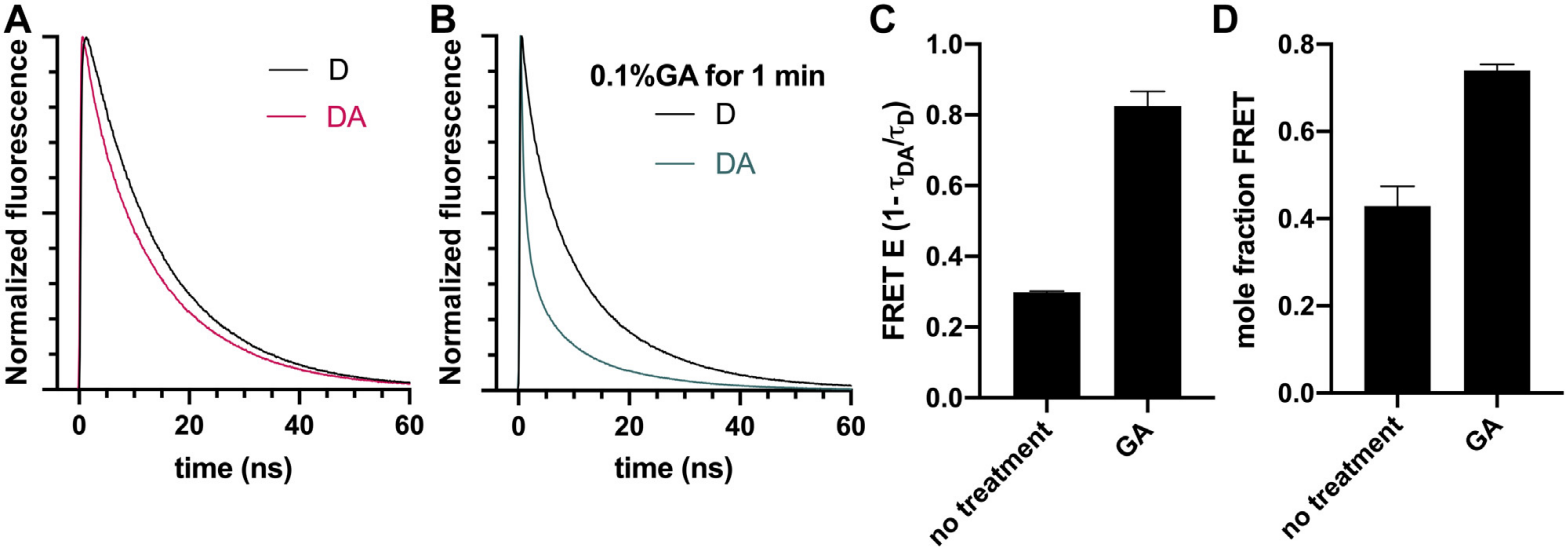
**TR-FRET detects the interacting-heads motif, and treatment with glutaraldehyde (GA) increases FRET.** Representative waveforms for donor-only (D) and donor–acceptor (DA) samples are shown (*A*) without GA treatment and (*B*) after GA treatment. (*C*) Effect of GA treatment on FRET efficiency (E, as calculated from amplitude-weighted average lifetimes τ of the D and DA waveforms), n = 3 per condition. (*D*) Changes in the mole fraction of the population participating in FRET.

Purified bovine cardiac HMM myosin was labeled with EDANS and dabcyl through the exchange of RLC labeled on the C-lobe by sulfhydryl reaction between an exogenous cysteine residue on the protein and maleimide on the label, as described in our previous work (Fig. 1*A*) (16). To minimize the donor-only population, the ratio of donor to acceptor was 1:3, leading to detectable FRET (Fig. 2*A*). Labeling did not significantly affect the actin association and disassociation (Fig. 1*B*), actin-activated ATPase activity (Fig. 1*C*), or single nucleotide turnover kinetics (Fig. 1*D*).

### Time-resolved FRET detects the closed state of HMM myosin

Time-resolved fluorescence of labeled HMM was best fit by a two-component model: 63% (±5%) of the donors were unaffected by FRET, having a lifetime identical to the donor-only sample, whereas the remaining donors showed substantial FRET, fitting to a Gaussian distance distribution having a center of 2.0 nm (±0.02) with a full-width of 1.4 nm (±0.02). We conclude that myosin molecules with the open conformation have a donor–acceptor distance that is too large to detect and that 43 ± 5% of the myosin molecules are in the IHM state (Fig. 2*D*). To verify that we are detecting the IHM, we used a variety of myosin effectors known to increase the population of either IHM or SRX myosin.

### The closed-state population increases with the trapping of IHM myosin by cross-linker

To test whether the FRET pair on opposite heads of HMM can measure IHM, labeled HMM was treated with glutaraldehyde, a cross-linker used to trap the IHM in electron microscopy studies (Fig. 2*B*) (3). We found that glutaraldehyde significantly increased FRET (Fig. 2*C*).

As a control, a donor-only sample was also treated with glutaraldehyde. Glutaraldehyde caused changes in EDANS fluorescence. Using the same two-state model as above to fit a fraction donor-only and a fraction participating in FRET, we found that the fraction participating in FRET increased from 43% (±5%) to 74% (±1%) with glutaraldehyde treatment (Fig. 2*D*). The value predicted if all myosin molecules are participating in FRET is 75%, based on the 1:3 donor to acceptor ratio. Thus, we conclude that, when treated with glutaraldehyde, nearly all the myosin molecules are in the IHM.

### Increasing ionic strength, seen to decrease SRX, did not change the closed-state population

**Figure 3:**
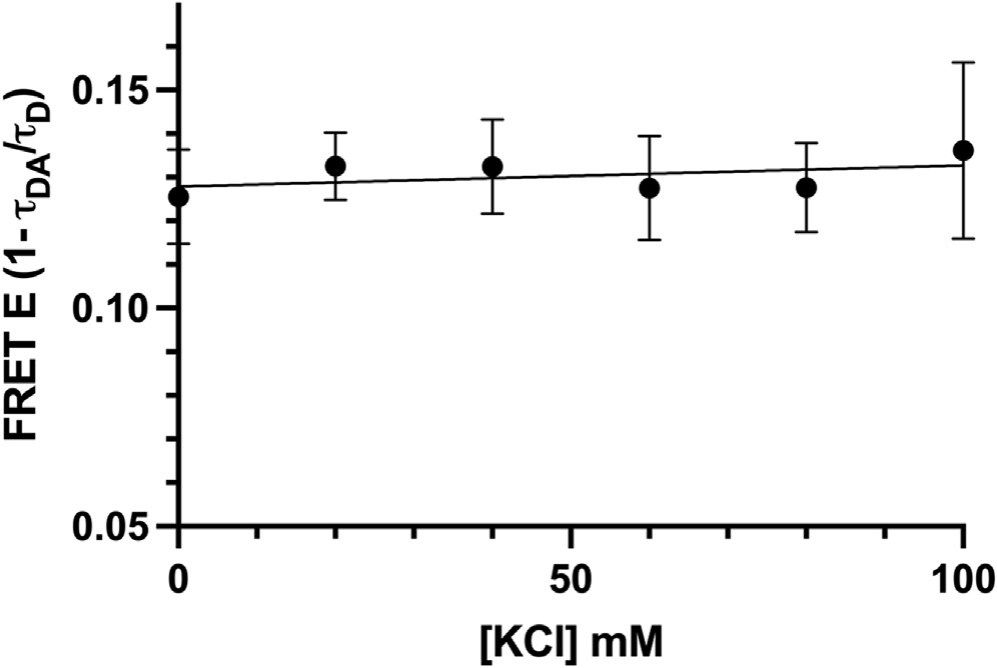
**Increasing ionic strength, seen to decrease super-relaxed state, did not change the closed population.** FRET efficiency (E as calculated from amplitude-weighted average lifetimes τ of D and DA waveforms) in range of 0 to 100 mM KCl, (n = 3 per condition, slope found to be not significantly different from zero). FRET, fluorescence resonance energy transfer.

To test whether the SRX and the IHM are identical, the same myosin was tested under conditions seen to increase the SRX. The ranges of ionic strengths (0–100 mM KCl) tested were previously reported to change the SRX population when measured by stopped-flow mant-ATP turnover kinetics (16). If the SRX and the IHM are identical, then FRET should increase. However, FRET in the samples did not change significantly, remaining at baseline (Fig. 3).

### Mavacamten, a stabilizer of the SRX, increases the closed-state population less than predicted

Mavacamten is a phase III drug used to treat hypertrophic cardiomyopathy. It has been shown to significantly decrease myosin activity, and electron microscopy has shown that cardiac myosin treated with the cross-linking version of mavacamten was entirely in the IHM state (16). Given this evidence, we predicted that a saturating dose of mavacamten should increase the FRET efficiency from 43% to 74%, if the IHM is fully populated, as predicted by the EM studies.

**Figure 4:**
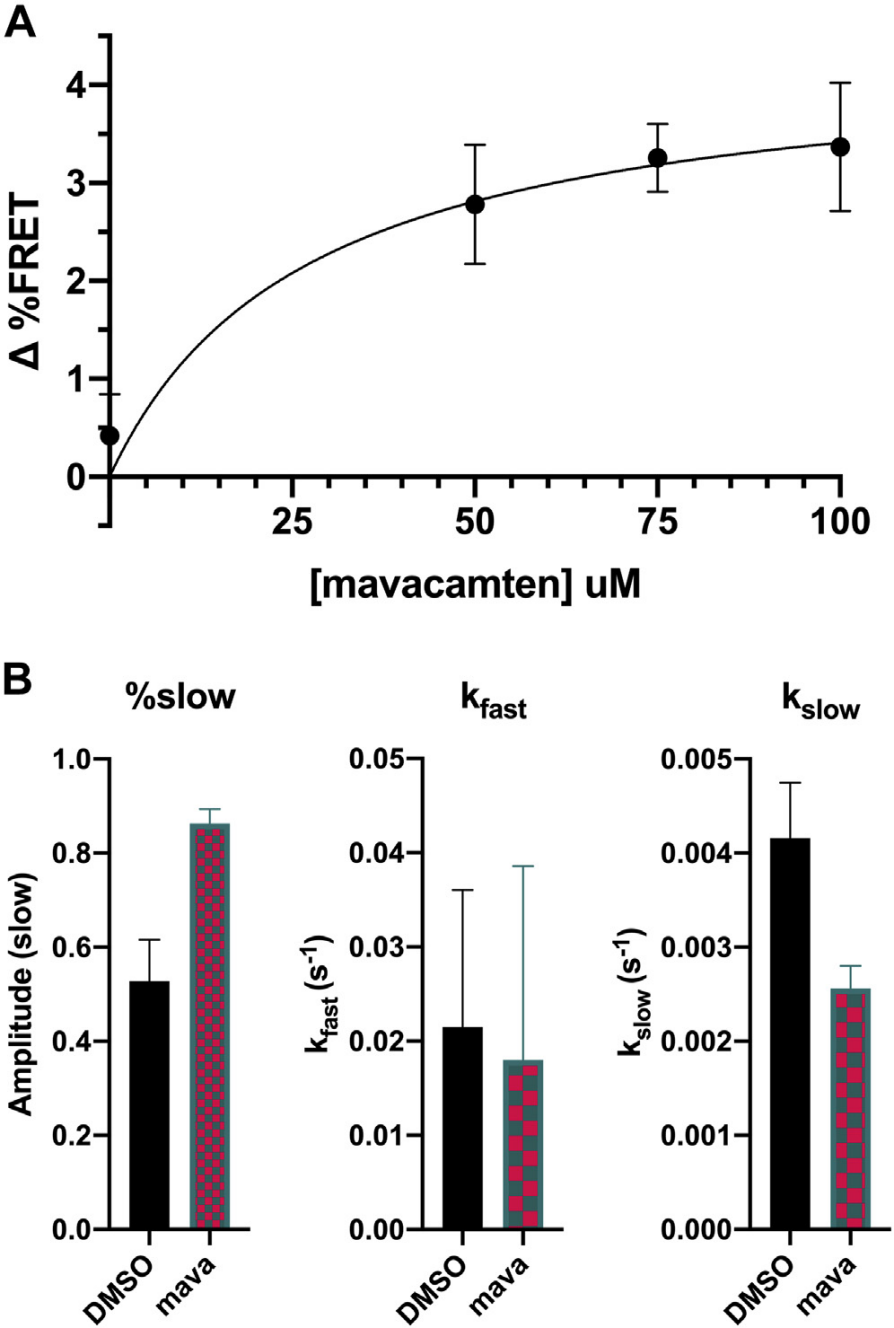
**Mavacamten causes a small increase in FRET but a much larger increase in super-relaxed state population in nucleotide turnover.***A*, Mavacamten dosage–response curve of FRET of 5 μM labeled heavy meromyosin (n = 3 per condition). *B*, Single nucleotide turnover of 0.2 μM labeled myosin with 10 μM mavacamten or 1% dimethyl sulfoxide (DMSO) mixed with 4 μM mant-ATP and chased with 2 mM ATP (n = 3 per condition). FRET, fluorescence resonance energy transfer.

Mavacamten was added to our FRET-labeled sample, and FRET did indeed increase, but only by about 3% (Fig. 4*A*), an order of magnitude less than predicted. Increasing mavacamten resulted in a hyperbolic response with a B_max_ of 4.3% and K_d_ of 27 μM. The 3% FRET change fits to a model with a 4% increase in the population participating in FRET. The labeled myosin gave the same robust response to mavacamten (33% increase in the population participating in the SRX) as unlabeled myosin in the fluorescent nucleotide turnover kinetics studies (Fig. 4*B*). When glutaraldehyde was added to samples treated with mavacamten, the population participating in FRET was not significantly different from samples treated with glutaraldehyde alone. Thus, consistent with previous findings, mavacamten increases the IHM population while also increasing the SRX population, but there is an order of magnitude greater effect on the SRX population.

## Discussion

Multiple forms of myosin, including mammalian cardiac myosin, appear to have a conserved mechanism of autoinhibition (3). Here we have directly observed the IHM of beta-cardiac myosin, using TR-FRET with randomly distributed donor- and acceptor-labeled RLC. We found that 43% (±5%) of the myosin molecules are in the IHM structural state (Fig. 2*D*). The SRX biochemical state, measured by fluorescent nucleotide turnover, has been found to vary from 40% to 70% in different beta-cardiac myosin of various species (16, 17, 18). We confirm that 53% (±9%) of the myosin was kinetically SRX in the stopped-flow mant-ATP chase experiments, which is comparable with the 43% of the structurally IHM myosin in the TR-FRET experiments. However, when we tested increasing ionic strength, seen to decrease the SRX population (16), FRET levels did not decrease. These results suggest that the structurally defined IHM is sufficient but not necessary for the low ATPase activity of the SRX.

The predicted distance in most reported models of the interacting heads motif of β-cardiac myosin, which are based on cryo-EM images of tarantula thick filaments (4, 13, 19), is around 5.0 nm, which is longer than the center distance seen by TR-FRET. In those cryo-EM studies, the region of lowest resolution is that of the RLC, suggesting that this area contains more disorder than the rest of the structure, so this discrepancy is not surprising (19). Other factors, such as environmental effects on the fluorescent molecules, may also contribute to this discrepancy.

We were unable to observe the open conformation of myosin with this FRET system, indicating that most of the heads on these myosin molecules spend most of their time at least 4 nm apart, as predicted by most models (13).

We tested whether the shorter distance corresponds to the IHM by treating myosin with glutaraldehyde, a cross-linker used to trap the IHM structure in myosin dimers for electron microscopy (3). We found that glutaraldehyde greatly increased FRET between donor- and acceptor-labeled RLC, confirming the IHM assignment.

In this study, we use HMM, a cleaved form of β-cardiac myosin that does not include the whole section of the tail region that is hypothesized to stabilize the IHM. In future, it will be important to use labeled RLC to measure the IHM in full-length myosin, as well as native thick filaments, which would also include accessory proteins that could stabilize IHM.

Given the effectiveness of mavacamten to inhibit beta-cardiac myosin contractility (20) and help patients with hypertrophic cardiomyopathy (21), we hypothesized that mavacamten protects the heart through sequestering of myosin heads in the inactive SRX biochemical state, which is outside of the mechanochemical cycle. Our laboratory and others have found that mavacamten potently increases the mole fraction of the SRX population (measured by nucleotide exchange) of β-cardiac myosin (16, 17). However, we observed that mavacamten did not greatly increase the mole ratio of the IHM state (measured structurally by FRET). Since our study detects the structural state of IHM under conditions (no freezing, no fixing) similar to those used previously to detect SRX, these results call into question the unconfirmed hypothesis that there is a one-to-one correspondence between the IHM structural state and the SRX biochemical state. Our results point to multiple structural states leading to SRX-like kinetics, one being the IHM and the other indistinguishable (by FRET) from the open conformation.

Based on our current results, we hypothesize that mavacamten does not fully trap cardiac myosin in the IHM. This may explain why it successfully treats hypertrophic cardiomyopathy without completely stopping heart contractions. Studies in cardiac S1, a cleaved single-headed version of myosin, showed that mavacamten primarily inhibits myosin by slowing the phosphate release and ADP release steps in the mechanochemical cycle, without head–head interaction (20). The slowing of these rates may also slow the single-turnover kinetics, leading to SRX-like rates. Our results show that the IHM-like structure leads to SRX-like kinetics but that the population of myosin in SRX can greatly exceed that in the IHM. Thus, there is a second (structurally distinct) population of SRX myosin having kinetics thought to be cardioprotective, but that does not require static sequestration *via* the IHM structure.

## Experimental procedures

### Protein preparations

β-Cardiac myosin was purified from bovine hearts obtained on wet ice from *Pel-Freez*, as described in Rohde *et al.* (22). This isolation takes advantage of the tendency of myosin thick filaments to associate and dissociate in buffers of various ionic strengths, and cycling through multiple times leaves a pure sample of myosin. The entire purification process was performed at 4 °C.

Unlabeled β-cardiac HMM was prepared as described in our previous work (22). Full-length bovine β-cardiac myosin was digested to HMM in 10 mM tris(hydroxymethyl)aminomethane (Tris), 600 mM KCl, 2 mM MgCl_2_, and 1 mM DTT (pH 7.5) with α-chymotrypsin (Sigma-Aldrich, 0.025 mg/ml final concentration) for 10 min at 25 °C, followed by addition of pefabloc (Roche, 5 mM final concentration), then dialyzed into 10 mM tris(hydroxymethyl)aminomethane (Tris) (pH 7.5) with 2 mM MgCl_2_, followed by Q-sephadex ion-exchange chromatography purification. Intact HMM samples without contaminants, as evaluated by SDS-PAGE, were pooled for experiments.

Recombinant bovine cardiac RLC, with a single reactive cysteine at position 105, was expressed in *Escherichia coli* and purified by inclusion body isolation followed by ion exchange chromatography as described in our previous work (22). We labeled the purified RLC with 10 molar excess of 5-((((2-Iodoacetyl)amino)ethyl)amino) naphthalene-1-sulfonic acid (IAEDANS, Invitrogen) or dabcyl C2 maleimide (AnaSpec) *via* a maleimide-sulfhydryl reaction overnight at 4 °C and then removed free dye by gel filtration chromatography. The labeled RLC was flash frozen in liquid nitrogen and stored at −80 °C until use.

Labeling efficiency was determined by dye absorbance and Bradford protein concentration assay, as well as liquid chromatography mass spectrometry. Labeling was confirmed using ultraperformance liquid chromatography-electrospray ionization mass spectrometry (UPLC-ESI MS). Unlabeled and fluorescently labeled RLC samples (≈50 mM) were buffer exchanged into 10 mM ammonium bicarbonate using Zeba desalting columns according to manufacturer's protocol (ThermoFisher Scientific). The sample was then run through a Waters C4 reversed-phase chromatography column with a gradient of water (0.1% formic acid) and acetonitrile (0.1% formic acid) (low organic to high organic over 26 min linear gradient), 0.4 ml/min into the Synapt G2 QTOF MS, which is scanned continuously from *m/z* 400 to *m/z* 2500.

Labeled β-cardiac HMM was prepared by first exchanging labeled RLC onto full-length myosin and then enzymatically digesting to HMM. Full-length myosin was mixed with 4 molar equivalents of labeled RLC in 1:3 EDANS-dabcyl (or unlabeled for the donor-only sample) ratio and 12 mM ethylenediaminetetraacetic acid (EDTA) and incubated for 10 min at 30 °C, causing the endogenous RLC to dissociate from myosin. The addition of 14 mM MgCl_2_ induces association of labeled RLC with myosin. After the RLC exchange, the labeled myosin was brought to 2 mM MgCl_2_ in its freezing buffer before digestion to HMM with α-chymotrypsin (Sigma-Aldrich, 0.025 mg/ml final concentration) for 10 min at 25 °C, followed by addition of pefabloc (Roche, 5 mM final concentration) and then dialyzed into 10 mM Tris pH 7.5 with 2 mM MgCl_2_. Digested HMM was purified by ion-exchange chromatography (GE HiPrep) and confirmed by SDS-PAGE prior to experiments.

### Time-resolved FRET measurements

TR-FRET waveforms were measured by time-correlated single-photon counting after excitation with a 381-nm subnanosecond pulsed diode laser (LDH-P-C-375B, PicoQuant). Emitted light was selected using a 440 ± 20-nm filter and detected with a PMH-100 photomultiplier (Becker-Hickl). Ten cycles of photon counting were measured and averaged per sample, and at least three biochemically distinct samples were tested. Mavacamten was added at the indicated concentrations, along with 2 mM magnesium ATP, and incubated for 10 min. Glutaraldehyde-treated samples (5 μM) in 10 mM MOPS, 30 mM KCl, 2 mM MgATP, and 1 mM DTT, pH 7.5, were treated with a 0.1% glutaraldehyde solution for 1 min, then quenched with 100 mM Tris (pH 7.5). The instrument response function was recorded from water.

TR-FRET data were then analyzed by fitting fluorescence decay waveforms by nonlinear regression analysis to a multiexponential decay, as described (22). The fluorescence decay can generally be described by a sum of exponentials:

(1) $$\text{F}\left(\text{t}\right)=\sum {{\text{A}}_{\text{i}}}_{\;}\;\text{exp}\left(-\text{t}/{\text{τ}}_{\text{i}}\right)$$

where τ_i_ is the fluorescence lifetime of the i-th component. FRET efficiencies were calculated with the amplitude (A)-weighted average lifetimes (τ) of the D and DA samples:

(2) $$E=1-\;\frac{\tau_{DA}}{\tau_{D}}$$

Glutaraldehyde treatment produced fluorescent signals with low intensity and short lifetimes, in both labeled and unlabeled samples, so the glutaraldehyde-treated waveforms were analyzed with the donor-only sample treated with glutaraldehyde. The number of donor-only lifetimes τ_i_ was determined first by fitting F_D_(t) to Equation 1, varying n from 1 to 4, choosing n as the smallest value needed to minimize χ^2^. The sample containing both donor and acceptor was fit to different structural models with the free parameters of mole ratio and center distance and full-width half-maximum of one (or more) Gaussian-distributed population. The model that most effectively minimized χ^2^ was chosen. The donor–acceptor waveform was also fit to a model in which the mole fraction of the population participating in FRET is allowed to vary:

(3) $$F_{DA}\left(t\right)=\;X_{D}\left(F_{D}\left(t\right)\right)+\left(1-X_{D}\right)\left(F_{DA}\left(t\right)\right)$$

where the mole fraction of donors participating in FRET is

(4) $$X_{FRET\;}=1-X_{D}$$

### Steady-state ATPase assay

The actin-activated MgATPase activity of purified cardiac myosin HMM or S1 was measured using an NADH-coupled assay performed at 25 °C in 10 mM Tris and 2 mM MgCl_2_ with 1 mM DTT (pH 7.5) in a 96-well clear plate. The reaction mix contained 0.2 μM HMM, varied actin concentrations, 0.2 mM NADH, 0.5 mM phosphoenol pyruvate, 2.1 mM ATP, 10 U/ml lactic acid dehydrogenase, and 40 U/ml pyruvate kinase. The conversion of NADH to NAD^+^ was measured on a SpectraMax 384 by monitoring absorbance at 340 nm wavelength for 30 min. The activity was calculated from the slope obtained by linear regression and divided by the concentration of catalytic sites in sample.

### Basal ATPase assay

Single-ATP turnover experiments with 2′- (or-3′)-O- (N-methylanthraniloyl) adenosine 5′-triphosphate (mant-ATP) were performed at 25 °C in 10 mM Tris, 25 mM potassium acetate, 4 mM MgCl_2_, 1 mM EDTA, and 1 mM DTT (pH 7.5). Steady-state fluorescence (total fluorescence intensity) was detected on an Applied Photophysics stopped-flow spectrophotometer capable of single-mix and sequential-mix experiments with water bath temperature control. Samples were excited at 280 nm with a xenon lamp and monochromator and were detected through a 400-nm long-pass filter. The single-mix dead time for this instrument is 1.3 ms. All buffers were filtered and then degassed for 30 min under high vacuum before use.

### Actin–myosin binding assay

Rabbit skeletal actin labeled with pyrene (0.5 μM) was mixed with HMM (labeled or unlabeled each at 1, 0.67, 0.5, 0.375, 0.28, 0.21, and 0.16 μM) in F-buffer (10 mM Tris pH 7.5, 3 mM MgCl_2_). Pyrene fluorescence (Ex = 365 nm, Em = 420 nm) was measured in a 96-well clear plate by a Molecular Devices Gemini EM microplate spectrofluorometer. ATP, 2 mM, was added for actin dissociation, and pyrene fluorescence was measured again.

## Data availability

Raw data will be shared upon request to corresponding author, D. D. T. (ddt@ddt.umn.edu).

## Conflict of interest

D. D. T. holds equity in, and serves as an executive officer for, Photonic Pharma LLC. These relationships have been reviewed and managed by the University of Minnesota. Photonic Pharma had no role in this study. All other authors declare that they have no conflicts of interest related to the contents of this article.
